# Supplementary material for: Morphologic changes of the no-touch saphenous vein as Y-composite versus aortocoronary grafts (CONFIG Trial)
Source: PLoS One. 2025 May 8;20(5):e0322176. doi: 10.1371/journal.pone.0322176 (PMC12061138; doi:10.1371/journal.pone.0322176)

**Supporting information**

**S1 Fig. Microscopic examination of the saphenous vein grafts**

Small segments of the saphenous vein grafts at proximal anastomotic end (Y-composite or aortocoronary anastomotic ends) were sampled just before the construction of the anastomosis and the intima-media thickness (IMT) of the saphenous vein was measured by microscopic examination.


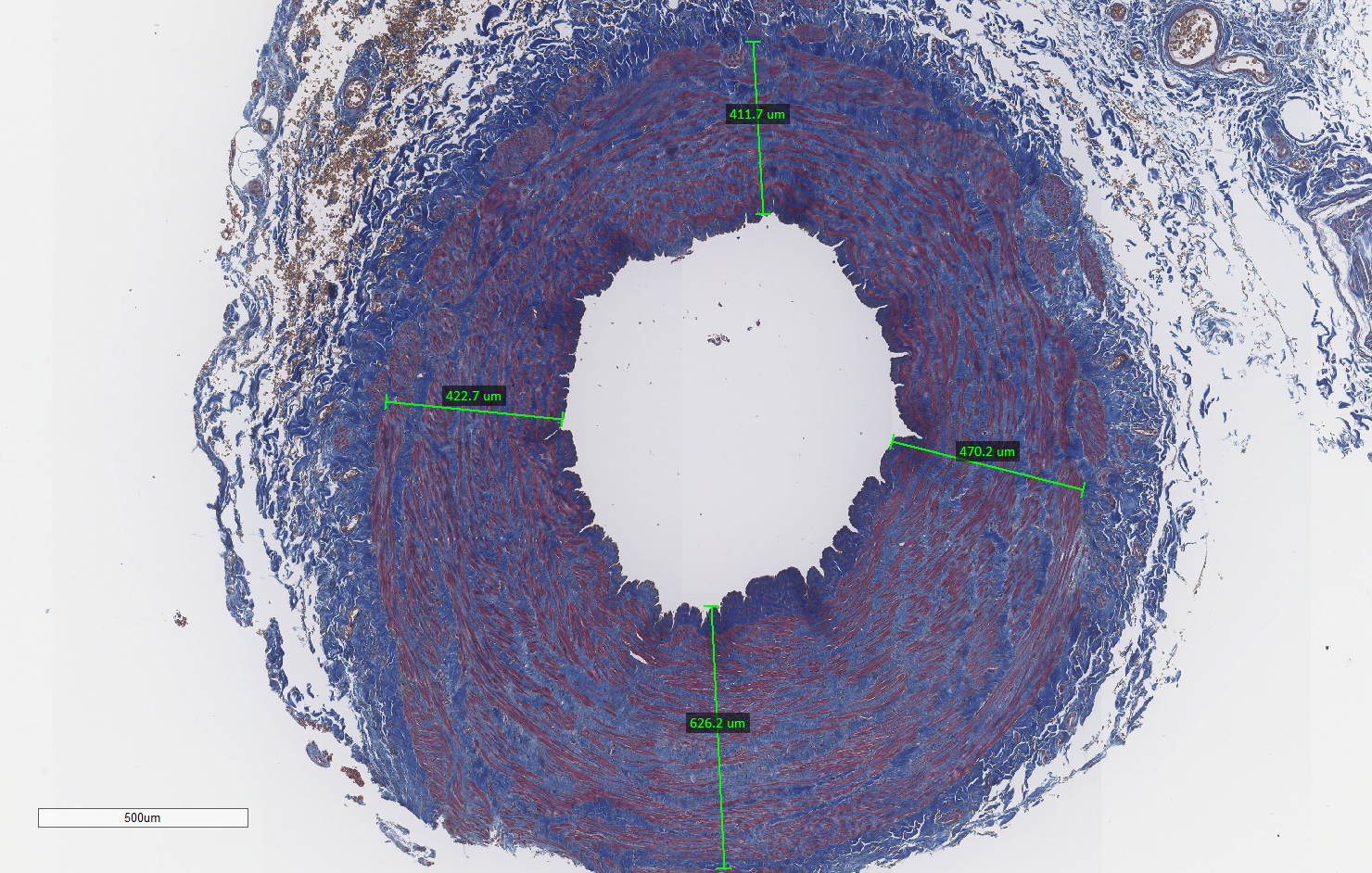

Supplement: S1 Fig — (DOCX) [file pone.0322176.s001.docx]
