# Supplementary material for: Morphologic changes of the no-touch saphenous vein as Y-composite versus aortocoronary grafts (CONFIG Trial)
Source: PLoS One. 2025 May 8;20(5):e0322176. doi: 10.1371/journal.pone.0322176 (PMC12061138; doi:10.1371/journal.pone.0322176)
Supplement: S1 Table — (DOCX) [file pone.0322176.s002.docx]

**Supporting information**

**S1 Table. Comparison of early postoperative angiographic patency rates between the 2 groups.**

| Variables | Total  (n = 50) | Composite group  (n = 25) | Aorta group  (n = 25) | *P* |
| --- | --- | --- | --- | --- |
| Overall | 99.5% (183/184) | 98.9% (93/94) | 100.0% (90/90) | >.99 |
| LITA | 100.0% (52/52) | 100.0% (26/26) | 100.0% (26/26) | - |
| SV | 99.2% (131/132) | 98.5% (67/68) | 100.0% (64/64) | >.99 |
| Anastomosed to LAD territory | 100.0% (42/42) | 100.0% (21/21) | 100.0% (21/21) | - |
| Anastomosed to LCX territory | 98.1% (51/52) | 96.3% (26/27) | 100.0% (25/25) | >.99 |
| Anastomosed to RCA territory | 100.0% (38/38) | 100.0% (20/20)^*^ | 100.0% (18/18) | - |
| Sequential anastomosis | 100.0% (82/82) | 100.0% (43/43) | 100.0% (39/39) | - |
| Terminal anastomosis | 98.0% (49/50) | 96.0% (24/25) | 100.0% (25/25) | >.99 |

^*^ 3 of 20 anastomoses showed competitive flow.

LAD, left anterior descending artery; LCX, left circumflex artery; LITA, left internal thoracic artery; RCA, right coronary artery; SV, saphenous vein.
