# Supplementary material for: Morphologic changes of the no-touch saphenous vein as Y-composite versus aortocoronary grafts (CONFIG Trial)
Source: PLoS One. 2025 May 8;20(5):e0322176. doi: 10.1371/journal.pone.0322176 (PMC12061138; doi:10.1371/journal.pone.0322176)
Supplement: S1 Study Protocol — (PDF) [file pone.0322176.s004.pdf]

Morphologic changes of the saphenous vein as Y-composite graft based on the left internal thoracic artery versus aortocoronary conduit for coronary artery bypass grafting: A prospective randomized controlled trial (CONFIG trial)

NCT 04782492

Trial Protocol v 1.2

Updated Jan 2, 2023

Protocol summary

**Trial name and number**	Morphologic changes of the saphenous vein as Y-composite graft based on the left internal thoracic artery versus aortocoronary conduit for coronary artery bypass grafting: A prospective randomized controlled trial (CONFIG trial)<br>ClinicalTrial.gov 04782492
**Objectives**	This randomized controlled trial is designed to directly compare 1-year morphologic changes of the no-touch saphenous vein as Y-composite (Composite group) versus aortocoronary (Aorta group) grafts in coronary artery bypass grafting (CABG).
**Design**	Prospective randomized controlled trial
**Study periods**	IRB approval date ~ December 31, 2023
**Subjects**	Patients who are scheduled to undergo primary isolated CABG
**Sample size**	Total 50 patients
**Methods**	Patients over 19 years of age who were scheduled to undergo primary isolated CABG for a multivessel disease on a nonemergency basis and in whom the use of left internal thoracic artery (LITA) and saphenous vein (SV) as bypass conduits was planned were assessed for eligibility for study enrollment. The exclusion criteria included (1) patients undergoing concomitant cardiac procedures including valve or aorta surgery, (2) patients in whom it was not feasible to use the LITA or the SV as a bypass conduit due to intrinsic problems with the vessels, (3) patients with severely atherosclerotic or calcified ascending aorta that precluded the use of aortocoronary anastomosis of the SV, (4) patients who had vasculitis, (5) patients with severe comorbidities that might limit the possibility of 1-year angiographic follow-up and (6) patients who declined to participate in the study.<br>Enrolled patients are randomly assigned to the Y-composite group (the Composite group) or aortocoronary group (the Aorta group) in a 1:1 manner. After surgery, early clinical outcomes and early postoperative angiographic patency are evaluated following the protocol. After discharge, all patients undergo regular postoperative follow-up. At 1 year after surgery, patients are evaluated with coronary angiography and intravascular ultrasound.
**Endpoints**	The primary endpoint of the CONFIG trial was intima-media thickness (IMT) measured by intravascular ultrasound (IVUS) on 1-year angiogram. The secondary endpoints were luminal diameter measured by IVUS on 1-year angiogram, 1-year angiographic graft patency, and clinical outcomes including all-cause mortality, cardiac death, target vessel revascularization, and reintervention at 1 year.

**Safety**	Postoperative complications including operative mortality, low cardiac output, bleeding reoperation, perioperative myocardial infarction, perioperative stroke, acute kidney injury, respiratory complications, postoperative atrial fibrillation, and mediastinitis were monitored for safety.
**Expectations**	This trial will provide the difference in the morphologic change of the no-touch SV according to the graft configuration, and help the patients and surgeons to choose optimal graft configuration for long-term patency of SV graft in CABG.

Study Protocol

1. Title

Morphologic changes of the saphenous vein as Y-composite graft based on the left internal thoracic artery versus aortocoronary conduit for coronary artery bypass grafting: A prospective randomized controlled trial (CONFIG trial)

2. Institutions

Seoul National University Hospital (101 Daehak-ro, Jongno-gu, Seoul, 03080, Korea)

3. Principal investigator and co-investigators

1) Principal investigators

Ho Young Hwang (Department of Thoracic and Cardiovascular Surgery, Seoul National University Hospital)

Jeehoon Kang (Department of Internal Medicine and Cardiovascular Center, Seoul National University Hospital)

2) Co-investigators

Yoonjin Kang (Department of Thoracic and Cardiovascular Surgery, Seoul National University Hospital)

Ji Seong Kim (Department of Thoracic and Cardiovascular Surgery, Seoul National University Hospital)

3) Sub-investigator

Suk Ho Sohn (Department of Thoracic and Cardiovascular Surgery, Seoul National University Hospital)

4. Sponsor

None.

5. Funding

None.

6. Study period

IRB approval – December 31, 2023

7. Study subjects

Patients who are scheduled to undergo primary isolated CABG

8. Background and Objectives

1) Background

Revascularization of the left anterior descending artery (LAD) with the left internal thoracic artery (LITA) has been recognized as the standard of care for coronary artery bypass grafting (CABG) because of its excellent long-term patency and survival benefit.[1] However, the grafting strategy for non-LAD target vessels is still controversial. Although many surgeons contend that multiple arterial grafting yields better outcomes than single arterial grafting,[2,3] multiple arterial grafting is used in only 3.9% to 34.2% of CABG patients in the North America and Europe.[4,5] In contrast, the saphenous vein (SV) has been the most commonly used conduit for several decades.[6,7] Although there has been concern regarding the high failure rates of SV grafts in the short- and long-term,[8,9] recent advances such as novel no-touch (NT) SV harvesting techniques and grafting strategies have shown improved graft patency rates for the SV.[10,11,12]

Regarding the grafting strategy of the SV in CABG, previous studies demonstrated favorable graft patency rates and negative remodeling of NT-SV when the SV was used as a Y-composite graft based on the in situ LITA.[12,13] However, whether the favorable findings for NT-SV composite grafts were due to the effect of using Y-composite grafting or to the NT harvesting technique could not be conclusively determined because these two modifications were adopted at the same time during the study period; no study has directly compared the results of SV grafts harvested using the same technique but different grafting strategies.

A randomized controlled trial entitled as 'Morphologic Changes Of the No-touch saphenous vein as Y-composite versus aortocoronary graFts In coronary artery bypass Grafting(CONFIG)' is designed to compare the 1-year morphologic features of NT-SV used as Y-composite grafts based on the in situ LITA with those of NT-SV used as aortocoronary grafts.

2) Hypothesis and Objectives

The present study is conducted to compare the 1-year morphologic features of NT-SV used as Y-composite grafts based on the in situ LITA with those of NT-SV used as aorto-coronary

grafts.

We hypothesize that the intima-media thickness (IMT) of the SV from aortocoronary configuration is higher than that of the SV from Y-composite configuration at 1-year angiographic follow-up and IVUS study.

9. Inclusion criteria, Exclusion criteria and Sample size calculation

1) Inclusion criteria

① Patients who are scheduled to undergo primary isolated CABG for a multivessel disease

② Patients over 19 years of age

③ Patients who are planned to use of LITA and SV as bypass conduits

④ Patients who or whose legal representative fill out a written consent form before the start of the clinical trial and patients who can comply with the clinical trial requirements

2) Exclusion criteria

① Patients with concomitant cardiac procedures including valve or aorta surgery

② Patients in whom it is not feasible to use the LITA or the SV as a bypass conduit due to intrinsic problems with the vessels

③ Patients with severely atherosclerotic or calcified ascending aorta that precludes the use of aortocoronary anastomosis of the SV

④ Patients who have vasculitis

⑤ Patients with severe comorbidities that may limit the possibility of 1-year angiographic follow-up

⑥ Patients who refused study enrollment

3) Sample size calculation

Based on previous studies, the reference IMT was estimated; 0.31±0.12 mm for the Y-composite configuration[13] and 0.43±0.09 mm for the aortocoronary configuration.[14,15] The study was designed to have 90% power to detect a significant difference in IMT between the 2 groups with a 2-sided type I error of 5.0%. Based on this power calculation, 17 patients were needed in each group. Allowing for a 30% dropout rate during the 1-year follow-up, recruitment of 50 patients (25 patients in each group) was determined to be necessary.

(1) Level of significance (α) = 0.05

(2) Type II error (β) = 0.10, power of the test = 90%

(3) Drop out rate = 30%

(4) Two-tailed test

H_0(null hypothesis): $\mu_1 = \mu_2$

H_1(alternative hypothesis): $\mu_1 \neq \mu_2$

(μ_1: mean IMT of SV graft of the study group on 1 − year IVUS,

μ_2: mean IMT of SV graft of the control group on 1 − year IVUS)

The calculation was performed using PASS Software(Power Analysis and sample size software: http://www.ncss.com), and the calculated formula is as follows.

$$n = \frac{\left(Z_{\frac{\alpha}{2}} + Z_\beta\right)^2 (\sigma_1{}^2 + \sigma_2{}^2)}{(\mu_1 - \mu_2)^2}$$

μ_1 = mean IMT of SV graft in the study group on 1 − year IVUS
μ_2 = mean IMT of SV graft in the control group on 1 − year IVUS
σ_1 = standard deviation of IMT of SV graft in the study group on 1 − year IVUS
σ_2 = standard deviation of IMT of SV graft in the control group on 1 − year IVUS

4) Subject recruitment plan

Subject recruitment will be performed for the patients who are scheduled to undergo isolated primary CABG and who are hospitalized at Seoul National University Hospital I after providing sufficient explanation and informed consents.

10. Methods

1) Detailed process of the study

Patients over 19 years of age who were scheduled to undergo primary isolated CABG for a multivessel disease on a nonemergency basis and in whom the use of LITA and SV as bypass conduits was planned were assessed for eligibility for study enrollment. After excluding the patients who meet the exclusion criteria and who refuse to participate, enrolled patients are randomly assigned to 1 of the 2 configurations in a 1:1 manner.

Operative strategies and Randomization process

Surgeries will be performed under standard median sternotomy, and off-pump CABG (OPCAB) will be the preferred grafting strategy during the study period. The in situ LITA will be

harvested using the skeletonization technique. The SV will be simultaneously harvested, preferentially from the lower leg, using a NT technique that retains perivascular soft tissue as previously described.[16] Randomization will be performed after the conduits are harvested without injury and it is confirmed that the intraoperative findings indicate that either of the grafting strategies are feasible. Web-based block randomization will be performed using randomly determined block sizes of 4 and 6. After randomization, the procedures will be performed according to the assigned grafting strategy. In the patients assigned to the Composite group, the Y-composite graft is constructed first by anastomosis of the distal end of the SV graft to the side of the LITA, and anastomosis of the LITA to the LAD territory is performed whenever necessary. The SV is then anastomosed to the diagonal branch, to the vessels in the left circumflex coronary artery(LCX) territory, and then to the vessels in the right coronary artery(RCA) territory as needed using a sequential anastomotic technique. In the patients assigned to the Aorta group, the SV is first anastomosed to the ascending aorta using the Heartstring III Proximal Seal System (MAQUET Holding B.V. & Co. KG, Rastatt, Germany). The LITA is then anastomosed to the target vessel in the LAD territory. Finally, the SV is anastomosed sequentially to the diagonal branch, to the vessels in the LCX territory and then to the vessels in the RCA territory as needed using a sequential anastomotic technique.

※ Randomization : Patients who meet the inclusion criteria and the exclusion criteria are randomly assigned to 1 of the 2 configurations in a 1:1 manner. Randomization is performed with a web-based block randomization method (https://mrcc.snuh.org/) with randomly determined block sizes of 4 and 6.

Histologic evaluation of the saphenous vein

Immediately after harvesting, the distal end of the SV, approximately 2mm in length, will be sampled and preserved as formalin-fixed paraffin-embedded tissue for histologic evaluation. The histologic evaluation is designed to compare the intima-media thickness (IMT) of the SV in the 2 groups as a baseline value of the IMT and as an indicator of the quality of the SV. The specimen will be divided into 3 sections perpendicular to the vessel lumen. Histologic evaluation will be performed using hematoxylin and eosin staining and Masson's trichrome staining. The IMT will be measured in 4 directions for each section, and the values obtained for the 3 sections will be averaged.

Postoperative process and follow-up plan

Patients are evaluated with early clinical outcomes and early postoperative angiographic measurements. After discharge, all patients undergo regular postoperative follow-up through the outpatient clinic at 3- to 6-month intervals and are interviewed by telephone for confirmation of their condition if the last clinic visit has not been conducted as scheduled. At postoperative 1-year, clinical outcomes and angiographic measurements are evaluated as scheduled. The measurement of IMT using IVUS will be simultaneously performed during 1-year angiographic evaluation.

Intravascular ultrasonographic (IVUS) study

The IVUS study will be performed after the completion of 1-year postoperative angiography in a standard fashion using an automated motorized pullback system (0.5 mm/s) with commercially available imaging catheters (Boston Scientific/SCIMED, Minneapolis, Minnesota, USA). The IVUS images will be acquired after the administration of 100 to 200 mg of nitroglycerin. In case of Y-composite grafting, the IVUS catheter will be advanced into the SV conduit connected to the mid-portion of the LITA and located at around 5 cm distal to the Y-anastomosis, and a pullback will be performed for the total length of 10 cm. To avoid the influence of suture materials, the areas 1 cm proximal and distal from the Y-anastomosis will be excluded from the analysis. In case of aortocoronary grafting, the IVUS catheter will be advanced into the SV conduit and located at around 5 cm distal to the aortocoronary anastomosis, and a pullback will be performed for the total length of 5 cm. The IVUS catheter will be also advanced into the LITA conduit and located at around 15 cm distal to the subclavian arterial origin, and an automated motorized pullback will be performed for the total length of 10 cm. To be a reference to the Y-composite group, the areas 1 cm distal from the aortocoronary anastomosis and the middle 2cm of evaluated LITA will be excluded from the analysis. The IVUS analysis will be performed by 1 independent experienced observer. Quantitative analyses of the IVUS data will be performed using computerized planimetry software (echoPlaque 3.0, Indec Systems Inc, Santa Clara, California, USA).

The lumen and vessel areas will be measured every 1 millimeter. All volumes will be calculated using the Simpson rule and then normalized for analyzed length. The area of intima-media (IM) will be calculated by subtracting the luminal area from the vessel area. The proportion of IM area to vessel area will be also calculated.

2) Outcome measures

1) Demographics: age, sex, height, weight, body mass index, body surface area, diagnosis,

operation name

2) Preoperative data

- Risk factors: diabetes mellitus, hypertension, smoking, dyslipidemia, stroke, chronic obstructive pulmonary disease, chronic kidney disease, chronic liver disease, coronary disease, peripheral vascular disease, history of cardiac surgery, New York Heart Association functional class, STS mortality risk, EuroSCORE II, SYNTAX score
- Electrocardiography
- Pulmonary function test
- Laboratory tests
- Echocardiographic measurements: LV ejection fraction, LV end diastolic dimension, LV end systolic dimension, regional wall motion abnormalities, etc.
- Coronary angiography : 1-vessel disease/2-vessel disease/3-vessel disease/left main disease, degree of stenosis for each branch

3) Operative data: number of anastomoses, target vessels, location of anastomosis, operative findings

4) Postoperative data

- Early clinical outcomes: op mortality, op morbidities (low cardiac output, bleeding reoperation, perioperative myocardial infarction, perioperative stroke, acute kidney injury, respiratory complications, postoperative atrial fibrillation, and mediastinitis)
- Echocardiographic measurements: LV ejection fraction, LV end diastolic dimension, LV end systolic dimension, regional wall motion abnormalities, etc.
- Coronary angiography : patency of bypass grafts, patency of distal anastomoses, presence of competitive flows for each anastomosis

5) 1-year follow-up data

- Clinical outcomes: all-cause mortality, cardiac mortality, major adverse cardiac events, reintervention, target vessel revascularization
- Echocardiographic measurements: LV ejection fraction, LV end diastolic dimension, LV end systolic dimension, regional wall motion abnormalities, etc.
- Coronary angiography : patency of bypass grafts, patency of distal anastomoses, presence of competitive flows for each anastomosis
- Intravascular ultrasound (IVUS) : intima-media thickness (IMT), lumen diameter (LD)

※ Primary clinical endpoint

Intima-media thickness (IMT) measured by IVUS on 1-year angiogram

※ Secondary clinical endpoint

Luminal diameter measured by IVUS on 1-year angiogram

One-year angiographic graft patency

Clinical outcomes including all-cause mortality, cardiac death, target vessel revascularization, and reintervention at 1 year

3) Differences from previous treatments and studies

Both of the configurations, Y-composite and aortocoronary, are commonly used in clinical practice, and have demonstrated good long-term outcomes in many previous studies.

When compared with previous studies, the present study is the first prospective randomized controlled trial comparing the morphologic change of SV graft using IVUS measurements.

4) Risks associated with the study

*** Predicted risks**

Because both configurations have proved their efficacy and safety by prospective and many retrospective studies, the predicted risks associated with the present study will be comparable to routinely performed CABG.

If any adverse event or risk occurs, providing the standard management for them will be sufficient.

5) Criteria for suspension and dropout

Patients who decline to participate in the trial are suspended or drop-out

6) Safety

Safety outcomes will be a composite of death from any cause and individual major postoperative complications including low cardiac output, bleeding reoperation, perioperative myocardial infarction, perioperative stroke, acute kidney injury, respiratory complications, postoperative atrial fibrillation, and mediastinitis.

All safety outcome measures will be assessed and recorded at designated time intervals by research personnel at the center. Data regarding every event will be reviewed and adjudicated by the principal investigator and sub-investigator.

7) Efficacy

Efficacy analysis will be performed for ITT (*Intention to treat*) group, and supplemental

analysis will be performed for PP (*Per protocol*) and *As treated* group. The primary endpoint will be concluded as 'significantly different' if both ITT and PP analyses confirm the significance of difference.

ITT group includes all study population. PP group is constituted from the ITT group after excluding the patients with major violation of the study protocol.

The null hypothesis is that the IMT of Y-composite group is comparable to that of aortocoronary group measured by IVUS on 1-year angiogram. The result for the primary endpoint will be presented with 95% two-sided confidence interval for mean difference between groups. The superiority test will be performed using a t-test which compared mean difference between groups under the two-sided significance level of 0.05.

For analysis of 1-year clinical outcomes, including all-cause mortality, cardiac death, target vessel revascularization, and reintervention, events will be counted at postoperative 1 year, and comparisons between the 2 groups will be made using the chi-square test and Fisher's exact test. A P value of < .050 will be considered statistically significant.

8) Schedules

① Study planning : March 1, 2021 – June 30, 2021

② Enrollment : July 8, 2021 – October 26, 2022

③ Data analysis and reporting : January 1, 2023 – December 31, 2023

11. Data monitoring and Safety committee

1) Monitoring committee

Ho Young Hwang (Department of Thoracic and Cardiovascular Surgery, Seoul National University Hospital)

Suk Ho Sohn (Department of Thoracic and Cardiovascular Surgery, Seoul National University Hospital)

2) Data and Safety Monitoring List

Operative mortality, low cardiac output, bleeding reoperation, perioperative myocardial infarction, perioperative stroke, acute kidney injury, respiratory complications, postoperative atrial fibrillation, and mediastinitis

3) Data and Safety Monitoring Periods

Safety outcomes will be monitored every 6 months.

4) Reporting drug adverse events, non-compliance, or unpredicted events

If any drug adverse event, non-compliance, or unpredicted event occurs, they must be reported within 15 working days from the date of recognition.

5) Withdrawal of the trial

If critical adverse events or complications associated with the trial occur and it is impossible to keep the trial going on, the trial will be withdrawed.

12. Ethics

The Investigators and all parties involved will conduct this study in adherence to the ethical principles based on Declaration of Helsinki, GCP, ICH guidelines and the applicable national and local laws and regulatory requirements. Relevant study documentation will be submitted to Ethics Committees of participating centers, according to local/national requirements, for review. Written approval of the study must be obtained locally before the study commences at each participating center. Once protocol amendments or consent form modifications are approved and implemented at the lead center, updated documents will be provided to participating centers. On completion of the study, the regulatory authorities will be notified that the study has ended.

13. References

1. Cameron A, Davis KB, Green G, Schaff HV. Coronary bypass surgery with internal-thoracic-artery grafts--effects on survival over a 15-year period. N Engl J Med. 1996;334:216-9.
2. Chikwe J, Sun E, Hannan EL, et al. Outcomes of Second Arterial Conduits in Patients Undergoing Multivessel Coronary Artery Bypass Graft Surgery. J Am Coll Cardiol. 2019;74:2238-2248.
3. Rocha RV, Tam DY, Karkhanis R, et al. Multiple Arterial Grafting Is Associated With Better Outcomes for Coronary Artery Bypass Grafting Patients. Circulation. 2018;138:2081-2090.
4. Gaudino M, Chikwe J, Falk V, Lawton JS, Puskas JD, Taggart DP. Transatlantic editorial: The use of multiple arterial grafts for coronary revascularization in Europe and North America. J Thorac Cardiovasc Surg. 2020;159:2254-2259.
5. D'Agostino RS, Jacobs JP, Badhwar V, et al. The Society of Thoracic Surgeons Adult Cardiac Surgery Database: 2018 Update on Outcomes and Quality. Ann Thorac Surg. 2018;105:15-23.
6. Favaloro RG. Saphenous vein autograft replacement of severe segmental coronary artery occlusion: operative technique. Ann Thorac Surg. 1968;5:334-9.
7. Schwann TA, Tatoulis J, Puskas J, et al. Worldwide Trends in Multi-arterial Coronary Artery

Bypass Grafting Surgery 2004-2014: A Tale of 2 Continents. Semin Thorac Cardiovasc Surg. 2017;29:273-280.

8. Goldman S, Zadina K, Moritz T, et al. Long-term patency of saphenous vein and left internal mammary artery grafts after coronary artery bypass surgery: results from a Department of Veterans Affairs Cooperative Study. J Am Coll Cardiol. 2004;44:2149-2156.

9. Lopes RD, Hafley GE, Allen KB, et al. Endoscopic versus open vein-graft harvesting in coronary-artery bypass surgery. N Engl J Med. 2009;361:235-244.

10. Souza DS, Johansson B, Bojo L, et al. Harvesting the saphenous vein with surrounding tissue for CABG provides long-term graft patency comparable to the left internal thoracic artery: results of a randomized longitudinal trial. J Thorac Cardiovasc Surg. 2006;132:373-8.

11. Samano N, Geijer H, Liden M, Fremes S, Bodin L, Souza D. The no-touch saphenous vein for coronary artery bypass grafting maintains a patency, after 16 years, comparable to the left internal thoracic artery: A randomized trial. J Thorac Cardiovasc Surg. 2015;150:880-8.

12. Kim KB, Hwang HY, Hahn S, Kim JS, Oh SJ. A randomized comparison of the Saphenous Vein Versus Right Internal Thoracic Artery as a Y-Composite Graft (SAVE RITA) trial: One-year angiographic results and mid-term clinical outcomes. J Thorac Cardiovasc Surg. 2014;148:901-7; discussion 907-8.

13. Hwang HY, Koo BK, Oh SJ, Kim KB. Morphologic changes of the saphenous vein Y-composite graft based on the left internal thoracic artery: 1-year intravascular ultrasound study. J Thorac Cardiovasc Surg. 2015;149:487-93.

14. Johansson BL, Souza DS, Bodin L, et al. Slower progression of atherosclerosis in vein grafts harvested with 'no touch' technique compared with conventional harvesting technique in coronary artery bypass grafting: an angiographic and intravascular ultrasound study. Eur J Cardiothorac Surg. 2010;38:414-9.

15. Taggart DP, Ben Gal Y, Lees B, et al. A Randomized Trial of External Stenting for Saphenous Vein Grafts in Coronary Artery Bypass Grafting. Ann Thorac Surg. 2015;99:2039-45.

16. Kim YH, Oh HC, Choi JW, Hwang HY, Kim KB. No-Touch Saphenous Vein Harvesting May Improve Further the Patency of Saphenous Vein Composite Grafts: Early Outcomes and 1-Year Angiographic Results. Ann Thorac Surg. 2017;103:1489-1497.
