# Supplementary material for: Morphologic changes of the no-touch saphenous vein as Y-composite versus aortocoronary grafts (CONFIG Trial)
Source: PLoS One. 2025 May 8;20(5):e0322176. doi: 10.1371/journal.pone.0322176 (PMC12061138; doi:10.1371/journal.pone.0322176)
Supplement: Attachment — Submitted filename: 2023 CONFIG final result_revision letter2.docx [file pone.0322176.s007.docx]

Re: PONE-D-24-50175

**Morphologic Changes of the No-Touch Saphenous Vein as Y-composite Versus Aortocoronary Grafts (CONFIG Trial)**

Dear Editors and Reviewers,

We have revised our manuscript entitled “**Morphologic Changes of the No-Touch Saphenous Vein as Y-composite Versus Aortocoronary Grafts (CONFIG Trial)”."** Followings are the point-by-point responses to the reviewers’ comments and the changes we have made;

**Reviewer #1:**

Comment 1

As the statistical reviewer I will focus on methods and reporting. The paper is well written, clear and with appropriate methods. The power calculations are also appropriate and there is broad balance between the groups. I only have a minor point to raise.

1) Can the authors clarify in the methods section how they planned to deal with missing data? were all the data complete? if yes, please state so. if not, why wasn't multiple imputation or another appropriate approach

Response 1

There were 1 missing data out of 25 patients in the Composite group and 3 missing data out of 25 patients in the Aorta group in terms of primary endpoint.

As Reviewer indicated, we additionally performed multiple imputation to deal with missing data, and revised the manuscript accordingly.

Change 1

Line 176

For the primary endpoint, multiple imputation was additionally performed to deal with missing data using Fully Conditional Specification (FCS) method.

Line 224

When further analysis was performed using multiple imputation to deal with missing data, the intima-media thicknesses of the SV grafts also demonstrated no difference between the groups (0.25 ± 0.06 mm vs. 0.24 ± 0.06 mm in Composite vs. Aorta groups, *P* =.76).

**Reviewer #2:**

Comment 1

This paper aims to compare 1-year morphologic changes of the no-touch saphenous vein graft as Y-composite(Composite group) versus aortocoronary(Aorta group) configurations in coronary artery bypass grafting. The paper is well structured in all parts. I suggest to revise the language and to add the following paper in the discussion: 10.31083/j.fbe1402012

Response 1

As Reviewer indicated, we cited the article by Jiritano et al in the manuscript.

Change 1

Line 271

A major concern of harvesting SV with the surrounding PVAT might be an increased risk of leg wound complication. Although it was beyond the scope of the present study, this caveat could be solved by various efforts including topical application of autologous platelet rich plasma [27].

In addition, references were renumbered as they appeared in the text.

Sincerely yours,

Ho Young Hwang

Professor

Department of Thoracic & Cardiovascular Surgery

Seoul National University Hospital

Seoul, Korea
